# The interaction between different types of activated RAW 264.7 cells and macrophage inflammatory protein-1 alpha

**DOI:** 10.1186/1748-717X-6-86

**Published:** 2011-07-22

**Authors:** Zhongshi He, Hui Zhang, Chunxu Yang, Yajuan Zhou, Yong Zhou, Guang Han, Ling Xia, Wen Ouyang, Fuxiang Zhou, Yunfeng Zhou, Conghua Xie

**Affiliations:** 1 Department of Radiation and Medical Oncology, Zhongnan Hospital, Wuhan University, 169, Donghu Road, Wuchang District, Wuhan, Hubei 430071, P.R. China; 2 Hubei Key Laboratory of Tumor Biological Behaviors, Wuhan University, Wuhan, 169, Donghu Road, Wuchang District, Wuhan, Hubei 430071, P.R. China

**Keywords:** Macrophage, MIP-1α, RAW 264.7 Cells, Classically Activated, Alternatively Activated, Chemotactic Ability

## Abstract

**Background:**

Two major ways of macrophage (MΦ) activation can occur in radiation-induced pulmonary injury (RPI): classical and alternative MΦ activation, which play important roles in the pathogenesis of RPI. MΦ can produce chemokine MΦ inflammatory protein-1α (MIP-1α), while MIP-1α can recruit MΦ. The difference in the chemotactic ability of MIP-1α toward distinct activated MΦ is unclear. We speculated that there has been important interaction of MIP-1α with different activated MΦ, which might contribute to the pathogenesis of RPI.

**Methods:**

Classically and alternatively activated MΦ were produced by stimulating murine MΦ cell line RAW 264.7 cells with three different stimuli (LPS, IL-4 and IL-13); Then we used recombinant MIP-1α to attract two types of activated MΦ. In addition, we measured the ability of two types of activated MΦ to produce MIP-1α at the protein or mRNA level.

**Results:**

Chemotactic ability of recombinant MIP-1α toward IL-13-treated MΦ was the strongest, was moderate for IL-4-treated MΦ, and was weakest for LPS-stimulated MΦ (p < 0.01). The ability of LPS-stimulated MΦ to secrete MIP-1α was significantly stronger than that of IL-4-treated or IL-13-treated MΦ (p < 0.01). The ability of LPS-stimulated MΦ to express MIP-1α mRNA also was stronger than that of IL-4- or IL-13-stimulated MΦ (p < 0.01).

**Conclusions:**

The chemotactic ability of MIP-1α toward alternatively activated MΦ (M2) was significantly greater than that for classically activated MΦ (M1). Meanwhile, both at the mRNA and protein level, the capacity of M1 to produce MIP-1α is better than that of M2. Thus, chemokine MIP-1α may play an important role in modulating the transition from radiation pneumonitis to pulmonary fibrosis *in vivo*, through the different chemotactic affinity for M1 and M2.

## Background

Radiation-induced pulmonary injury (RPI) can occur during radiotherapy for thoracic cancer and limits the radiation dose that can be applied. Although the histopathological features of RPI have been well documented, its pathogenesis has not been elucidated. Many types of inflammatory cells are involved in RPI, but pulmonary macrophages (MΦ) are the most prominent [1]. Different populations of activated MΦ can arise in response to distinct stimuli. When stimulated by lipopolysaccharide (LPS) and/or IFN-γ, the classically activated MΦ (M1) is generated, which secretes high levels of proinflammatory cytokines and mediators [2], and expresses inducible NO synthase (iNOS) [3]. M1 may enhance the microbicidal activity of MΦ and is closely associated with radiation pneumonitis. The amount of MΦ in the lung increases quickly after irradiation [2]. The second population of activated MΦ is alternatively activated MΦ (M2) that arises in the presence of the cytokines IL-4, IL-13, glucocorticoids, or TGF-β. M2 upregulates the expression of mannose receptors [4], decreases the antigen-presenting capability of MΦ, and shows high arginase 1 activity [3]. Arginase 1 can contribute to the production of ECM by catalyzing the formation of polyamines and collagen, overexpression of which improves pulmonary fibrosis. Excessive IL-4 and the related M2 have been observed in radiation pulmonary fibrosis (RPF) [2].

A variety of inflammatory cells play significant roles in RPI, and chemokines also have non-redundant roles of recruiting MΦ and other effector cells to the sites of inflammatory injury [4]. Chemokines, especially macrophage inflammatory protein-1α (MIP-1α, also known as CCL3) and related CC-chemokines, act as signal transducers in inflammatory injury, and perform important regulatory functions [5]. MIP-1α is thought to arise mainly from MΦ and epithelial cells in the lung. Different activated MΦ have different behavior related to MIP-1α secretion. M1 stimulated by LPS and IFN-γ promotes MIP-1a-generation, while IL-4 and IL-10 inhibit MIP-1a production of MΦ induced by LPS or IL-1β [6,7]. MIP-1α, which possesses strong chemotactic affinity for MΦ, is a critical MΦ chemoattractant in murine wound repair [8,9].

The hypothesis of a perpetual cascade of cytokines leading to RPI is a reasonable explanation [10]. However, the hypothesis does not specify which cell or cytokine dominates in the cascade response. The mechanism of the transition from radiation pneumonitis to RPF also is unknown, as is whether the chemotactic affinity of MIP-1α is different for distinct activated MΦ. We speculate that MIP-1α arises mainly from M1, while its chemotactic affinity toward M2 is stronger than for M1. The interaction between MIP-1α and MΦ in different activated states may play a crucial role in regulating the transition from radiation pneumonitis to RPF. By constructing classically and alternatively activated models of MΦ induced by different stimuli (LPS, IL-4 and IL-13), the interaction between MIP-1α and different activated MΦ was studied *in vitro *to investigate the pathogenesis of RPI.

## Materials and methods

### Macrophage culture

The murine MΦ cell line RAW 264.7 was obtained from the China Center for Type Culture Collection (CCTCC) at Wuhan University, and grown in DMEM supplemented with 10% heated-inactivated FCS, 2 mmol/L L-glutamine, and 100 U/mL penicillin/streptomycin (GIBCO) at 37ºC in a humidified incubator of 5% CO_2_. For some experiments, cells were starved, which means that cells were washed with phosphate-buffered saline (PBS) and incubated in DMEM supplemented with 100 U/mL penicillin/streptomycin for 12 h, but without 10% heated-inactivated FCS or 2 mmol/L L-glutamine. Cells between passages 5 and 20 were used in this study.

### Experimental design

**Figure 1:**
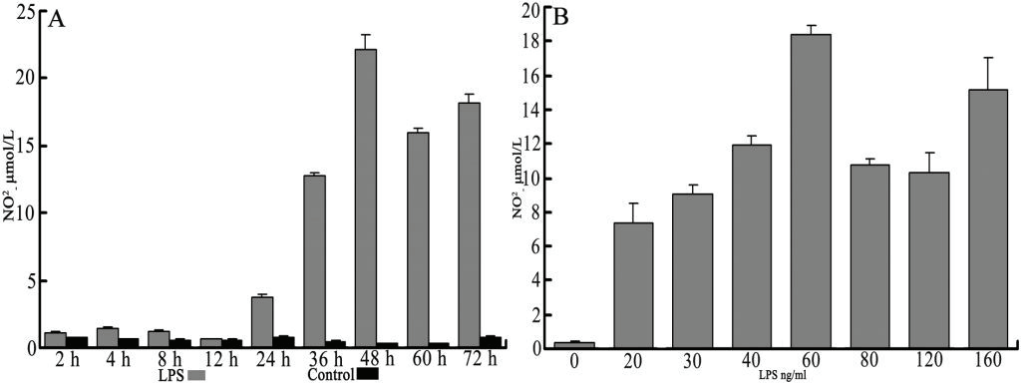
**NO production of RAW 264.7 cells stimulated by LPS**. **A**. RAW cells were exposed to either 0 ng/mL or 30 ng/mL LPS. At scheduled time points, the cell supernatant was collected for determination of NO_2_^- ^with Griess reagent. **B**. RAW cells were exposed to LPS for 48 h at different concentrations, then NO_2_^- ^was measured in the same way as in A. Values are averages ± SD of two independent experiments each done in triplicates; (**) indicates p < 0.01, (one way ANOVA).

**Figure 2:**
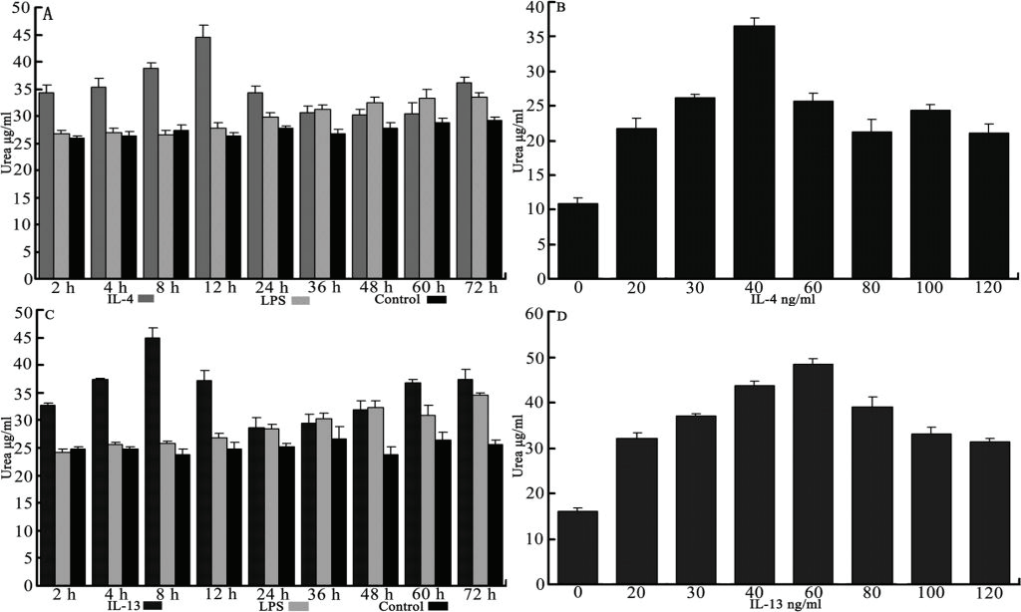
**Urea production of RAW 264.7 cells by IL-4 or IL-13**. RAW cells were exposed to 0 ng/mL, 30 ng/mL LPS, 30 ng/mL IL-4 (see Figure 2A) or 30 ng/mL IL-13 (see Figure 2C). At scheduled time points, the cells were collected for urea determination using a microplate method. RAW 264.7 cells were exposed to IL-4 for 12 h (see Figure 2B) or IL-13 for 8 h (see Figure 2D) at different concentrations, then urea was measured. Values are averages ± SD of two independent experiments each done in triplicates; (*) indicates p < 0.05, (**) indicates p < 0.01 (one way ANOVA).

Cells were plated in 24-well plates (for nitrite [NO_2_^-^] or urea measurements) at 5 × 10^5 ^cells/well. When the cells fully adhered after starvation for 12 hours, they were exposed to 30 ng/mL LPS (Sigma), IL-4 (PeproTech), or IL-13 (PeproTech), respectively. At the scheduled time points (see Figures 1A, 2A, C), the supernatant from the cells stimulated by LPS was collected for NO_2_^- ^measurement using the colorimetric Griess reaction [11]; cells stimulated by IL-4 or IL-13 were gathered to for urea measurement using a microplate method [12]. The best incubation time was determined by the preceding time points. Cells were plated and starved in the same way again, then exposed to LPS, IL-4, or IL-13 at seven different concentrations (see Figures 1B, 2B, D). After incubation, measurement of NO2^- ^was done for LPS-stimulated samples and measurement of urea was done for IL-4- or IL-13-treated samples to determine the best concentration for stimulus.

Cells were then plated in a culture flask at 5 × 10^5 ^cells/mL × 6 mL, for the chemotaxis assay, or in 60-mm dishes at 5 × 10^5 ^cells/mL × 3 mL for measurement of protein expression of MIP-1α from the cell supernatant, or for detection of MIP-1α mRNA in the cells. Optimal concentrations of LPS, IL-4, or IL-13, as determined by the earlier experiments, were used to determine the best times.

### Measurement of nitric oxide

The production of NO was measured by determining NO_2_^- ^in the culture supernatants using the colorimetric Griess reaction. Aliquots (60 μL) of cell supernatant were combined with an equal volume of Griess reagent [1% sulfanilamide (Alfa Aesar)/0.1% N-(1-napthyl) ethylenediamine (International Laboratory USA) -- each in 2.5% H_3_PO_4_] in a 96-well plate at room temperature for 10 min, and the absorbance at 550 nm was measured with a Multiscan plate reader (Genios, Tencan). Absorbance measurements were averaged and converted to μmol/L of NO_2_^- ^per well using a standard curve of sodium nitrite.

### Determination of arginase activity

Arginase activity was determined according to a microplate method with slight modification. After incubation for the scheduled time, the cells were rinsed with PBS, then lysed with 300 μL of 0.5% Triton X-100 that contained protease inhibitors (Sigma). After shaking for 30 min at room temperature, the lysate was mixed with 400 μL of 25 mmol/L Tris-HCL (pH 7.4) and 100 μL of 10 mmol/L MnCl_2_. The arginase was activated by heating for 10 min at 56ºC. Arginine hydrolysis to urea was conducted by addition of 50 μL of 0.5 mol/L L-arginine (pH 9.7) to 50 μL of the activated lysate, followed by incubation at 37ºC for 60 min. The reaction was stopped with 800 μL of H_2_SO_4 _(96%)/H_3_PO_4 _(85%)/H_2_O(1/3/7, v/v/v). Urea concentration was measured at 550 nm after addition of 50 μL of 9% (w/v) a-isonitrosopropiophenone (Tokyo Chemical Industry Co. LTD) dissolved in 100% ethanol and heating at 100ºC for 45 min. A standard curve was created using two-fold dilutions of urea (1.25 μg/mL to 640 μg/mL) following by mixing with the stop reagent and then heating.

### Chemotaxis assay

**Figure 3:**
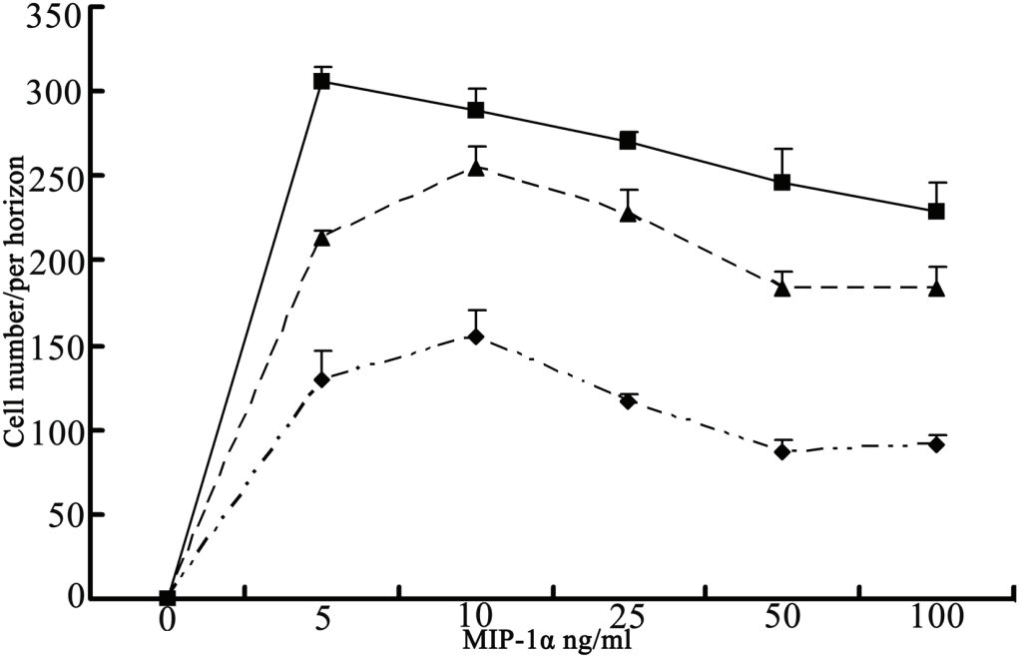
**Recombinant MIP-1α as a potent chemoattractant for MΦ *in vitro***. Cells were exposed to 60 ng/mL LPS for 48 h, 40 ng/mL IL-4 for 12 h, or 60 ng/mL IL-13 for 8 h, followed by cell collection. MΦ chemotaxis was measured in a Transwell chamber with rMIP-1α at several concentrations. Results are expressed as cell number/horizon under a light microscope (250 times) Values are averages ± SD done in triplicates; Significant difference (p < 0.01) of chemotactic ability was obvious for different activated states of MΦ (one way ANOVA).

The ability of rMIP-1α (PeproTech) to promote MΦ chemotaxis was measured with a 24-well Transwell chamber (Sigma). When the MΦ in the culture flask was stimulated, it was washed twice with PBS and suspended in DMEM at a concentration of 5 × 10^5 ^cells/mL. A series of MIP-1α or DMEM alone (negative control) (see Figure 3) were placed in the bottom wells of the chemotaxis chamber and 8- μm thick polycarbonate filters were placed on top of the wells. MΦ suspensions (200 μL) were placed on the top of wells and the chamber was incubated at 37ºC for 120 min. The filters were removed and nonmigrating cells (facing the top wells) were gently washed off with PBS and then air-dried. After staining MΦ with 150 uL of crystal violet, cell counts were determined using a light microscope to compare the strength of the chemotactic affinity.

### MIP-1α measurement by ELISA

Extracellular immunoreactive MIP-1α was measured by ELISA using a commercial kit (R&D) according to the manufacturer's instructions. Sample absorbance was measured with a Multiscan plate reader (Genios, Tencan) at a wavelength of 450 nm. The sample concentration was measured using a standard curve.

### Real-time quantitative PCR of MIP-1α

Total cellular RNA was extracted using Trizol according to the manufacturer's instructions. Then RNA was reverse-transcripted into cDNA using reverse-transcriptase (Toyobo). For amplification by PCR, the forward primer for MIP-1α was CTCCCAGCCAGGTGTCATT, and the reverse primer was GGCATTCAGTTCCAGGTCAG. The forward primer for β-actin was CCGTGAAAAGATGACCCAG, and the reverse primer was TAGCCACGCTCGGTCAGG. The PCR conditions were as follows: 95ºC, 45 sec; 60ºC, 15 sec; 72ºC, 45 sec for 40 cycles. Amplification was terminated by 10 min at 72ºC. For data analysis, the comparative threshold cycle (CT) value for β-actin was used to normalize loading variations in the real-time PCRs. ΔΔCT value then was obtained by subtracting the control ΔCT values from the corresponding experimental ΔCT values. The ΔΔCT values were compared with the control by raising two to the ΔΔCT power.

### Statistical analysis

Statistical analyses of data were conducted using one-way analysis of variance (ANOVA). Statistical significance was established at p < 0.05. The software used for statistical analysis was SPSS 13.0 (SPSS, Inc., Chicago, IL).

## Results

### Expression of macrophage enzyme activity

To obtain activated states of MΦ, MΦ was stimulated by LPS, IL-4, and IL-13, and then the activated states were evaluated by measuring iNOS and arginase activity. M1 induced by LPS expressed specific iNOS activity, while M2 stimulated by IL-4 or IL-13 showed particular arginase1 activity. Therefore, the magnitude of iNOS or arginase activity was chosen to reflect the strength of classically or alternatively activated states of MΦ.

Experimental results demonstrated that, compared with iNOS activity of quiescent MΦ, the activity in MΦ increased significantly after MΦ was stimulated by LPS (30 ng/mL) for 12 hours (p < 0.01), and peaked at 48 hours (see Figure 1A). When stimulated with various concentrations for a fixed time (48 h), MΦ induced by 60 ng/mL LPS expressed the greatest iNOS activity (see Figure 1B). Compared with arginase activity of quiescent and LPS-stimulated MΦ, arginase activity was increased significantly when MΦ was treated by IL-4 (30 ng/mL) within 24 hours (p < 0.01) or by IL-13 (30 ng/mL) within 12 hours (p < 0.01). The quiescent and LPS-stimulated MΦ also expressed arginase activity. In comparison with quiescent MΦ, the MΦ stimulated by LPS for 36 hours resulted in an increase of arginase expression (p > 0.05), but significantly less than the activity resulting from MΦ stimulated by IL-4 within 24 hours, or MΦ stimulated by IL-13 within 12 hours (see Figures 2A, C). When stimulated with different concentrations at a fixed time, MΦ induced by 40 ng/mL IL-4 or 60 ng/mL IL-13 showed the greatest arginase activity (see Figures 2B, D). Thus, the optimal conditions were stimulation of classically activated MΦ with 60 ng/mL LPS for 48 hours, stimulation of alternatively activated MΦ with 40 ng/mL IL-4 for 12 hours, and stimulation of alternatively activated MΦ with 60 ng/ml IL-13 for 8 hours.

These results provide further information about the factors involved in arginase activity from alternative macrophages. In contrast with a previous report of urea production from different activated MΦ [10], the present results showed that urea production of the cells produced a bell-shaped response with both IL-4 and IL-13 at different stimulation times or concentrations (see Figure 2). This difference was attributed to the experimental conditions that were repeatedly explored in the pre-experimental phase, and represents a change in arginase activity of RAW 264.7, indicating that stimulation time and concentration of the stimulus both significantly affect enzyme activity.

### Chemotactic ability of MIP-1α toward activated macrophages

A difference in the chemotactic ability of MIP-1α for different activated MΦ was verified. This difference was reflected in two ways. First, chemotactic ability was distinct for different activated states of MΦ (p < 0.01). Chemotactic ability of MIP-1α toward IL-13-treated MΦ was the strongest, was moderate for IL-4-treated MΦ, and was weakest for LPS-stimulated MΦ. Second, the peak concentration of MIP-1α for different activated MΦ also was different, with a peak concentration for IL-13-stimulated MΦ of 5 ng/mL, but a peak concentration for IL-4- and LPS-stimulated MΦ of 10 ng/mL (see Figure 3).

### Comparison of macrophages producing MIP-1α

**Figure 4:**
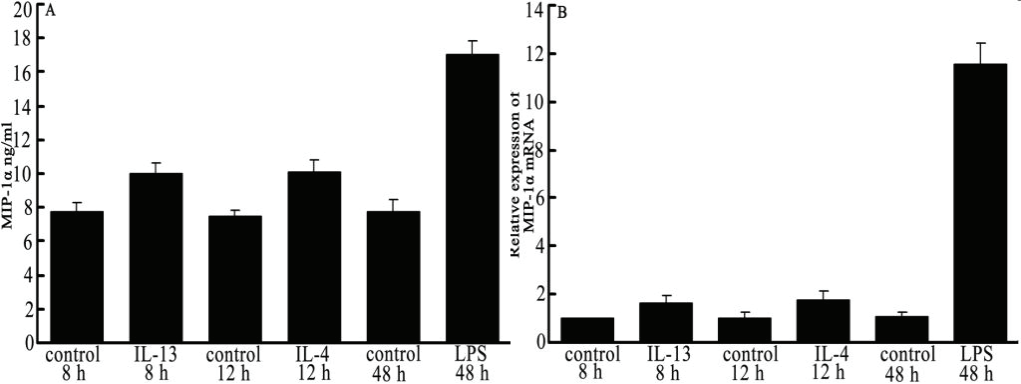
**Induction of MIP-1α expression in RAW 264.7 cells**. **A. **RAW cells were exposed either to 60 ng/mL LPS for 48 h, 40 ng/mL IL-4 for 12 h, or 60 ng/mL IL-13 for 8 h, followed by culture supernatant collection. Supernatant MIP-1α was assayed by ELISA. **B. **RNA was extracted from RAW cells treated as shown in A. MIP-1α mRNA levels were quantified using real-time RT-PCR, with an 8h control group. β-actin was used as an internal control. Calculation of fold values is described in Materials and Methods. Values are averages ± SD of two independent experiments each done in triplicates; (**) indicates p < 0.01 (one way ANOVA).

The capacity of MIP-1α production for different activated MΦ varied. MIP-1α production of quiescent MΦ at different time points was not statistical different (p > 0.05) at the mRNA or protein level. At the protein level, MIP-1α expression from cell supernatants was determined by ELISA. The ability of LPS-stimulated MΦ to secrete MIP-1α was significantly stronger than that of IL-4-treated or IL-13-treated MΦ (p < 0.01). Compared with untreated quiescent MΦ, the MΦ stimulated by IL-4 or IL-13 produced lower levels of MIP-1α secretion (see Figure 4A). At the mRNA level, MIP-1α expression from cells was determined by RT-PCR. The ability of LPS-stimulated MΦ to express MIP-1α mRNA also was stronger than that of IL-4- or IL-13-stimulated MΦ (p < 0.01) (see Figure 4B). Therefore, we conclude that at the level of either protein or mRNA, MΦ stimulated by LPS was able to express MIP-1α significantly better than MΦ stimulated by IL-4 or IL-13.

## Discussion

The interaction between chemokines and macrophages is complex, which significantly affects macrophage biological activity. Through experiments *in vitro*, we discovered that the chemotactic ability of MIP-1α toward M2 is significantly stronger than that for M1, while the capacity of M1 to produce MIP-1α is better than that of M2.

However, little information existed about whether a difference exists in the chemotactic ability of MIP-1α for different activated MΦ. Several groups have reported there is a preferential attraction of certain subsets of lymphocytes by human MIP-1α [13,14], MIP-1α is a potent chemoattractant for MΦ. By chemokine binding to cell surface CC chemokine receptors of MΦ, which belong to the G-protein-coupled receptor superfamily, the G-protein complex can induce Ca^2+ ^from extracellular and smooth endoplasmic reticulum influx into cytoplasm [15]. An increase in Ca^2+ ^in cytoplasm is necessary for MΦ migration. The results of our experiments indicate that the chemotactic ability of MIP-1α for M2 is significantly stronger than that for M1. LPS could rapidly inhibit expression of CC chemokine receptors by reduction of CCR1 mRNA levels in monocytes [16]. A distinct stimulus leading to differences in the properties and numbers of CC chemokine receptors in activated MΦs may contribute to the chemotactic ability disparity of MIP-1α for activated MΦ.

And there are mininal effective and maximal concentrations for human MIP-1α's chemotaxis. Human MIP-1α was found to chemoattract NK cells *in vitro*, and maximal activity was obtained at a concentration of 100-1000 ng/ml [17,18]. Our results may confirm a similar conclusion. At the concentration range of 8-18 ng/ml, MIP-lα shows maximum chemotactic activity for different activated macrophages.

Many cells, especially MΦ, can express low levels of MIP-1α constitutively, which can be induced or inhibited by regulators. The same regulator may exert an opposite effect on different cells. For example, IL-4 and IL-10 inhibit MIP-1α production of MΦ stimulated by LPS or IL-1β, while IL-4, IL-10, INF-γ, and IL-1β all induce vascular smooth muscle cells to produce MIP-1α [19]. Our experiments indicated that, at the mRNA and protein level, the ability of MΦ stimulated by LPS to secrete MIP-1a is significantly greater than that of MΦ stimulated by IL-4 or IL-13. Thus, the ability of M1 to produce MIP-1α is better than that of M2. The ability of M2 induced by IL-4 or IL-13 to produce MIP-1α is only slightly enhanced when compared to the control group, which seems to contradict IL-4 inhibition of LPS-induced MIP-1α secretion. This phenomenon may result from a difference in the original activated states of MΦ.

Different activated MΦ in RPI are induced by distinct cytokines generated by damaged cells after γ-ray irradiation of the lung. Classical activation of macrophages was originally reported to require both TNF-α and IFN-γ [20]. Bacterial endotoxin LPS was chosen as a stimulus for murine MΦ cell line RAW 264.7 cells to generate M1 in this study because LPS (a Toll-like receptor agonist) stimulates MΦ in an autocrine manner to induce both TNF and IFN-β and activate MΦ [21]. IFN-γ, LPS, and IFN-γ +LPS are weak, moderate, and strong inducers of iNOS activity, respectively, in *in vitro *experiments [22], so single stimulus LPS was best at inducing M1, when compared to other single inducers.

The M2 designation encompasses cells with differences in their biochemical and physiological activity [23]. People have attempted to further subdivide this type of MΦ, but a way to classify them further has not been developed. When stimulated by IL-4 and/or IL-13, MΦ can develop into alternatively activated (M2a). M2 can be further subdivided into those induced by immune complexes (ICs) and LPS or IL-1b (M2b) or those induced by IL-10, TGF-b, or glucocorticoids (M2c). However, one researcher [24] proposed that M2b and M2c belonged to a subtype of activated macrophages that required two stimuli to induce their anti-inflammatory activity. In our experiment, we select M2a as the alternative activated subtype because it is involved in injury repair and has been studied extensively.

Previous studies often have used a fixed-dose stimulus acting for a fixed time to generate activated MΦ [25]. Measuring enzyme activity of biomarkers iNOS and arginase 1 can reflect the strength of the biological activity of activated MΦ. Our study suggests that the biological activity of activated MΦ is different when induced by stimuli at different doses for different times. Therefore, the conditions that produce the optimal activation of MΦ *in vitro *must be investigated. The results of our experiments also show that M1 expresses arginase activity that is significantly weaker than that of M2a. Results of a previous study also demonstrated that arginase expression could be triggered by IL-4 and IL-10 as well as by detoxified LPS, while IFN-γ induced only NO synthesis in macrophages *in vitro *[26].

In conclusion, our data indicate that the chemotactic ability of MIP-1α for M2 is significantly stronger than for M1, while the capacity of M1 to produce MIP-1α is better than that of M2. RPI is a multi-cell and multi-cytokine-mediated cascading event, many cytokines such as TNF-α may play an important role in the process of RPI [27], but they could not completely explain its pathogenesis. The important roles of macrophages at different stages of RPI and the interactions between macrophages and chemokines may mean that chemokines could be key factors in the pathogenesis of RPI through chemotactic disparity of different cells, or even different subtypes of the same cell. Blocking the expression of MIP-1α or inhibiting its chemotactic ability could control the degree of repair *in vivo*, which may be a promising method of preventing RPI. Studies are continuing to examine the interactions between different activated MΦ and MIP-1α in an RPI mouse model, and their role in the pathogenesis of RPI.

## Competing interests

The authors declare that they have no competing interests.

## Authors' contributions

ZH and HZ contributed significantly to study design and concept. ZH, CY and YZ (Yajuan Zhou) contributed to manuscript writing and study coordinator. YZ (Yong Zhou) and GH contributed to statistical analysis. LX, WO and FZ contributed significantly to the acquisition of data and optimization of treatment plans. YZ (Yunfeng Zhou) and CX contributed to final revision of manuscript. All authors read and approved the final manuscript.
